# Cell-Free Extracts of the Ginseng Soil Bacterium *Pseudomonas plecoglossicida* Promote Suppression of Resistance of American Ginseng (*Panax quinquefolius*) to Root Rot Caused by *Ilyonectria mors-panacis*

**DOI:** 10.3390/biology13090671

**Published:** 2024-08-29

**Authors:** Paul H. Goodwin, Tom Hsiang

**Affiliations:** School of Environmental Sciences, University of Guelph, 50 Stone Road East, Guelph, ON N1G 2W1, Canada; thsiang@uoguelph.ca

**Keywords:** genomics, ginsenosides, ginseng replant disease, immunosuppressor, soil bacterium

## Abstract

**Simple Summary:**

Ginseng replant disease is associated with root rot caused by the soil-borne fungus, *Ilyonectria mors-panacis*, which is believed to be due to the combination of the pathogen with unknown host-related factors found in soil after harvesting ginseng. One candidate for the host-related factor was described in a prior report where methanol extracts from previous ginseng soil increased root lesion sizes during *Ilyonectria mors-panacis* infections associated with suppression of ginseng disease defense gene expression. As treatment of ginseng with ginseng root extract did not have these effects, it was unclear what was the source of this bioactivity in soil. This study showed that a soil bacterium, *Pseudomonas plecoglossicida*, isolated from soil previously used for ginseng production, can convert ginseng root compounds in culture into compounds able to increase infections by *Ilyonectria mors-panacis*, demonstrating a possible way that a host factor of ginseng replant disease is produced by microbial conversion of root materials in previous ginseng soils.

**Abstract:**

A prior report showed that soil previously planted with American ginseng (*Panax quinquefolius*) contained compound(s) which could reduce ginseng resistance to root infection by *Ilyonectria mors-panacis*, and this was not found in extracts from ginseng roots or soils not previously planted with ginseng. However, the origin of this ginseng-related factor in ginseng soils is unknown. An isolate of *Pseudomonas plecoglossicida* obtained from soil where *P. quinquefolius* had been harvested grew more in culture media when ginseng root extract was included, indicating the use of compounds in the extract as nutrients. Treatment with cell-free extracts from media containing ginseng root extracts where *P. plecoglossicida* had been cultured resulted in root lesions caused by *I. mors-panacis* being significantly larger than roots treated with fresh media containing root extract or with cell-free media inoculated with the same bacterial isolate without root extract. Levels of ginsenosides in the media decreased over time with incubation. Genome sequencing revealed that the bacterium had genes homologous to those reported for ginsenoside metabolism, which can release sugars for microbial growth. Thus, a ginseng soil bacterium, *P. plecoglossicida*, can create compound(s) suppressive to root rot resistance, similar to that found in soils previously planted with ginseng, indicating that the activity suppressing root rot resistance in soil previously planted with ginseng may be of microbial origin, utilizing compounds from ginseng roots.

## 1. Introduction

Ginseng replant disease is the unsuccessful cultivation of ginseng in the same field that was previously used for ginseng production and is associated with plant death due to root rot caused by *Ilyonectria mors-panacis* [1]. Ginseng can remain mostly disease-free in soil not planted previously with ginseng but will then experience major losses from root rot when replanted with ginseng at the same site. Westerveld and Shi [1] hypothesized that ginseng replant disease could be due to a build-up of pathogen inoculum from the first crop, a change in the soil microbiome increasing pathogens, an alteration of soil physiochemistry, and/or the accumulation of allelochemicals, such as aliphatic acids, phenolic acids, ginsenosides, or chemically/biologically transformed ginsenosides. They concluded that the most likely cause of ginseng replant disease is a combination of root rot by *I. mors-panacis* and unknown host-related factors in the soil following ginseng harvesting. 

One effect of ginseng cultivation is a modification of soil bacterial diversity [2]. For instance, cultivation of Asian ginseng (*Panax ginseng*) decreased soil bacterial diversity over six years, but while the Gammaproteobacteria decreased, Acidiobacteria and Solibateres increased [3]. Another case is the decrease in soil bacterial diversity with *P. ginseng* cultivation over four years, but while Actinobacteria and Proteobacteria increased, Proteobacteria and Acidiobacteria decreased [4]. For American ginseng (*Panax quinquefolius*), bacterial diversity decreased in soils following planting, with a number of bacterial species having biodegradation function becoming less abundant, such as species of *Methylibium*, *Sphingomonas*, *Variovorax*, and *Rubrivivax* [5]. These studies show that bacterial populations are clearly affected by ginseng cultivation. 

A key factor that determines the soil microbiome of plants is the rhizosphere effect, which is the growth enhancement of soil microorganisms primarily due to release of root exudates, mucilage, and root debris into the soil [6]. Ginseng root exudates contain a variety of compounds, such as toxic diisobutyl phthalate, that can affect the soil microbiome, reducing the abundance of *Arthrobacter*, *Burkholderia*, *Rhodanobacter*, and *Sphingobacterium* [4]. Ginseng root exudates also contain ginsenosides, which have been reported to be secreted from roots of *P. quinquefolius* at rate of 25 µg/day per plant, with the ginsenoside types in root exudate being the same as those in the roots [7]. Ginsenosides can also be released into soil from root debris, such as dead root cap cells, since all parts of the *P. ginseng* plant contain ginsenosides [8]. Application of 10 mg/L total ginsenosides of *P. ginseng* to soil generally lowers soil bacterial diversity, richness, and abundance, but some groups of bacteria increase in abundance [9]. Application of *Panax notoginseng* root exudates or a mixture of the ginsenosides Rg1  +  Rb1  +  Rd caused similar soil microbiome changes affecting 57 soil bacterial genera, and bacteria showing increased abundance in soil had a greater ability for growth to be promoted in culture by Rg1 + Rb1 + Rd along with the ability to use them as carbon sources for growth [10]. This was believed to be due to bacterial glycosyl hydrolases releasing sugars attached to ginsenosides as nutrients. In contrast, bacteria showing decreased abundance in ginsenoside-treated soil mostly had growth suppressed in culture with media containing the ginsenosides, and were less able to use Rg1  +  Rb1  +  Rd as carbon sources.

Deglycosylation of ginsenoside by bacteria involves the enzymatic removal of sugars at the C3 (or C6) and C20 positions [11]. A wide range of ginseng root and soil bacteria have enzymes for ginsenoside deglycosylation. One example is a β-glucosidase-producing bacterium, *Sphingomonas* sp. isolate ZY-3, isolated from *P. ginseng* soil that could hydrolyze Rb1 into CK by removing the outer glucose from C20, producing Rd, and then removing the outer and inner glucoses from Rd at C3, producing F2 and CK, respectively [12]. Another example is a glycoside oxidoreductase-producing *Rhizobium* sp. isolate GIN611 from *P. ginseng* soil that deglycosylated CK by oxidization to remove the second glucose at C20 to create the completely deglycosylated (*S*)-protopanaxadiol PPD (S) [13]. It appears there are many ginseng soil bacteria that can cause ginsenoside transformation in soil by microbial deglycosylation. 

Treating American ginseng roots with extracts of ginseng soil previously planted with ginseng increased root rot by *I. mors-panacis*, while treating with extracts of ginseng roots or soil not planted with ginseng did not [14]. The ginseng soil extract mentioned above also suppressed expression of several jasmonate-regulated genes in *P. quinquefolius* roots during infection with *I. mors-panacis* compared with roots treated with water or ginseng root extract, indicating that the innate immune system was suppressed. It appears that ginseng immunosuppressive compounds in soil previously planted with ginseng come from ginseng roots, but the original ginseng root compounds do not have activity against the ginseng innate immune system. Thus, one possibility is that they are created by microbial modification in soil. For example, compounds such as deglycosylated ginsenosides are not found in roots but may be found in non-sterile soil previously planted with ginseng. 

This work investigated whether bacteria could be isolated from soil previously used for ginseng cultivation that were able to convert compounds in ginseng root extract into compounds capable of increasing root rot caused by *I. mors-panacis*. A bacterial isolate with this ability was examined for changes in ginsenosides while it grew in media with *P. quinquefolius* root extract, and its genome was examined to determine whether there were genes for enzymes potentially involved in ginsenoside transformation. 

## 2. Materials and Methods

### 2.1. Biological Materials 

*Ilyonectria mors-panacis* isolate IMP.ND4Z15 was originally isolated from infected roots of *P. qiunquefolius* grown in non-replanted soil from Norfolk County, Ontario [15]. For the following experiments, the isolate was grown on PDA for 4 weeks in the dark at 22 °C. Three-year-old *P. qiunquefolius* roots, obtained from commercial ginseng gardens in non-replanted soil in Norfolk County, were rinsed with tap water and stored in dark plastic bags at 4 °C. 

### 2.2. Bacterial Isolation from Soil and Identification of Pseudomonas plecoglossicida 

Soil (100–200 g) was collected from seven former ginseng gardens in the Simcoe area (0.08, 1, 3, 5, 10 and 25-year-old post-harvest replanted soil) in September and October 2017 and stored at 4 °C. One gram of each soil sample was mixed with 100 mL dsH_2_O and placed on a shaker for 72 h at 175 rpm. Serial dilutions of 1:10, 1:100, and 1:1000 were prepared from the samples, and 100 μL of each dilution was spread onto LB agar. After 48 h, the number of colonies and colony types were assessed. A single colony of each colony type was then transferred to LB agar and grown for 48 h. For storage, bacterial cells were suspended in sterile 10% glycerol (Fisher Scientific, Toronto, ON, Canada) and placed at −80 °C.

Selected bacteria from single colonies were cultured on LB agar for 24 h, and DNA was extracted using isoamyl alcohol [16]. A single colony of the bacterial culture was suspended in 100 μL dsH_2_O and then 100 μL 24:1 chloroform/isoamyl alcohol (Sigma-Aldrich, Oakville, ON, Canada) was added to the suspension. The suspension was gently mixed by inversion and then centrifuged at 16000 rpm for 5 min at 4 °C. The upper phase of the solution was removed for PCR. PCR for the 16S rRNA [17] was carried out in 20 μL reactions containing 1.7 μL 10 mM buffer (BioBasic, Markham, ON, Canada), 2 μL 10 mM Mg^2+^, 0.34 μL 25 mM dNTP (BioBasic), 1 μL each of primers 27f (5′-GAGAGTTTGATCCTGGCTCAG-3′) and 1495r (5′-CTACGGCTACCTTGTTACGA-3′), 0.25 μL 5 U *Tsg* polymerase (Bio Basic), and 8 μL of bacterial DNA extract. PCR conditions were 94 °C for 3 min, followed by 35 cycles of 94 °C for 30 s, 55 °C for 1 min, 72 °C for 1 min, and then, one cycle at 72 °C for 10 min. The PCR product was then sent to the University of Guelph Laboratory Services for sequencing. The sequence was used as a query in BLASTN against the nr/nt database at NCBI (https://www.ncbi.nlm.nih.gov, accessed 29 August 2018). The matches with 100% query coverage and over 98.65% nt identity were used for species identification [18].

### 2.3. Genome Sequencing and Assembly of Pseudomonas plecoglossicida

*Pseudomonas plecoglossicida* isolate 17-08R was cultured on LB agar for 24 h at 30 °C, and the cells were then harvested and DNA extracted following Clarke et al. [19] with modifications. Agar was washed off bacterial cells with 2 mL phosphate-buffered saline (PBS). The suspension was centrifuged at 12,000 rpm for 5 min. The pellet was re-suspended in 200 μL PBS and 25 μL of (20 mg/mL) proteinase K (Sigma-Aldrich) was then added to the suspension, which was vortexed for 15 s. The mixture was then incubated at 70 °C for 10 min and gently mixed with an equal volume of phenol–chloroform and centrifuged at 12,000 rpm for 10 min at 4 °C. The supernatant was mixed with an equal volume of isoamyl alcohol and centrifuged at 12,000 rpm for 10 min at 4 °C. The supernatant was removed and 1:10 volume sodium acetate (3 M, pH 5.2) and 3 volumes ethanol (95%) were added. After incubation overnight at −20 °C, a pellet was obtained by centrifugation at 12,000 rpm for 10 min. The pellet was then washed with 1 mL 70% ethanol and dissolved in 100 μL of PCR H_2_O. DNA quality and quantity were determined using a NanoDrop Lite Spectrophotometer (ThermoFisher, Mississauga, ON, Canada). The DNA sample was sent to Québec Genome Centre (http://www.genomequebec.com, accessed 29 August 2018) for genome sequencing using 150 bp paired-end sequencing with an Illumina HiSeq X Ten (Illumina, San Diego, CA, USA). The raw sequenced reads were assembled into contigs and scaffolds using Velvet (https://www.ebi.ac.uk/~zerbino/velvet, accessed 29 August 2018), SOAPdenovo (http://soapdenovo2.sourceforge.net, accessed 29 August 2018), and Abyss (http://www.bcgsc.ca/platform/bioinfo/software/abyss, accessed 29 August 2018) assemblers with odd value k-mers ranging from 19 to 101. The assembly with the highest N50 value from among the three programs and different k-mers was selected. The N50 was the weighted median average scaffold length, which means that a scaffold of this length had 50% of all the sequenced bases in shorter scaffolds and 50% in larger scaffolds. From genome assemblies, genes were predicted using AUGUSTUS 3.3.1 (https://github.com/Gaius-Augustus/Augustus, accessed 29 August 2018).

For genomic comparisons, 14 genomes of *P. plecoglossicida* and a genome of *P. entomophila* with predicted genes or genes predicted using AUGUSTUS were obtained from the NCBI nr database (Table 1). Reciprocal comparisons of the predicted genes from each genome were made using Standalone BLASTN (BLAST v2.6.0+ [20] with an e-value cutoff of 1 × 10^−3^ and the output format set to tabular format.

### 2.4. Phylogeny of Pseudomonas plecoglossicida

A phylogram between the isolates was created based on the partial 16S rRNA sequence (AM905851.1) and the sequences of *rpoB* (HE577797.1), *rpoD* (HE577793.1), and *gyrB* (HE577791.1), which had previously been used for taxonomic analysis among 26 *Pseudomonas* isolates by Mulet et al. [22]. The sequences were used as queries in a BLASTN search of each bacterial genome. The closest match for each gene from each genome was collected and aligned by gene using MUSCLE [23], and the alignments were concatenated and used to produce a maximum-likelihood phylogram using RAxML version 8.0.0 [24]. The resulting phylograms were viewed using MEGA 6 [25].

Homologs were obtained using reciprocal best hit (RBH) analysis between all pairings of genomes, using BLASTN. The sum of matching nucleotide identities (nident) between the query and subject genomes was then divided by the summed lengths of the aligned total predicted sequences from a self-match of the query genome. The resulting percent identity matrix was used to construct a neighbor-joining (NJ) phylogram using the R package APE (analyses of phylogenetics and evolution) [26].

### 2.5. Selected Gene Predictions from the Genomes of Pseudomonas plecoglossicida 

Protein sequences predicted to be involved in altering ginsenosides were obtained from the NCBI databases (https://www.ncbi.nlm.nih.gov, accessed 27 September 2018). The search terms ‘ginsenoside transformation’, ‘metabolism of ginsenoside’, and ‘transform ginsenoside’ were used to obtain putative glycoside hydrolase protein sequences (Supplemental Table S1). The search terms were ‘ginsenoside hydrolyzing’ to obtain a β-d-xylosidase protein sequence, ‘(pseudomonas [Organism]) AND rhamnosidase)*’* to obtain a rhamnosidase protein sequence, and ‘(pseudomonas[Organism]) AND α-L-arabinofuranosidase ‘ to obtain an arabinofuranosidase protein sequence (Supplemental Table S2). The search terms were ‘ginsenoside aglycons’ to obtain outer membrane protein sequences (Supplemental Table S3), and ‘deglycosylation of ginsenosides’ to obtain glycoside oxidoreductase and TAT-pathway signal protein sequences (Supplemental Table S4). The protein sequences were used as BLASTP queries against the predicted proteins from each of the 14 genomes of *P. plecoglossicida* and a genome of *P. entomophila* (Table 1). 

For putative glycoside hydrolase and glycoside oxidoreductase proteins, the sequences were submitted to the CAZyme database dbCAN (http://www.cazy.org/,) accessed 27 September 2018) to identify the family of CAZyme, and InterPro (https://www.ebi.ac.uk/interpro/, accessed 27 September 2018)) to identify functional domains. Secretion of the proteins was predicted using the program SignalP version 4.1 g for classically secreted proteins which have a signal peptide. To predict whether secretion might occur through a non-classical secretion system (i.e., lacking a signal peptide), the predicted proteins were examined using SecretomeP 1.0h with a minimum 50% neural network (NN) score cutoff.

### 2.6. Ginseng Root Extract 

Dried methanol extract was obtained from 3-year-old *P. quinquefolius* roots using a method modified from Dai and Orsat [27]. Washed *P. quinquefolius* roots (20 g) were macerated with a mortar and pestle, transferred to a 200 mL flask, and 60 mL 80% methanol was then added. The solution was shaken overnight at 175 rpm. The mixture was vacuum filtered through No. 4 qualitative filter paper (Whatman, Maidstone, UK), and the solution was evaporated under vacuum at 40 °C. The dried residue was stored at 4 °C, and 200 mg was then solubilized in 1 mL of sterile distilled (sd) H_2_O and then filtered through a 0.22 μm membrane (Whatman).

### 2.7. Bacterial Growth and Ginsenoside Transformation

Single colony isolates of soil bacteria were grown on LB agar for 24 h, and the bacterial colonies were suspended in lysogeny liquid (LL) broth (1 g/L yeast extract, 0.5 g/L NH_4_Cl, 1 g/L K_2_HPO_4_, 0.5 g/L KH_2_PO_4,_ 0.25 g/L MgSO_4,_ pH 8) [28] and adjusted to 0.20 to 0.22 OD600. Then, 15 µL of the bacterial suspension was added to 15 mL LL broth or LLG broth (LL broth plus 48 mg/mL ginseng root extract prepared as described above). The broths were incubated for 20 days at 30 °C, shaking at 175 rpm. Bacterial growth was measured at OD600 using a spectrophotometer (SmartSpec Plus, Bio-Rad, Hercules, CA, USA) at 0, 1, 2, 3, 4, 5, 8, 10, 14, 15, 17, 19, and 20 dpi (days post inoculation). A standard curve of OD600 versus CFU/mL was created using serial dilution (10^5^ to 10^9^), measuring OD600 and counting the numbers of colonies from serial dilutions on LB agar. The formula generated was as follows: logCFU/mL = 9e + 10^9^ (OD600) + (3e + 10^8^). 

To identify ginsenosides in the broth, bacteria were grown as above, and LL or LLG broth was harvested at 0 (just prior to inoculation), 5, 10, 15, and 20 dpi. The media were centrifuged at 10,000 rpm, the supernatant was passed through a 0.22 μm membrane filter (Whatman), and the filtrate was mixed with an equal volume of 80% methanol (Fisher Scientific). Following evaporation under vacuum at 40 °C, dry residue was solubilized in 1 mL of sd H_2_O and filtered using 0.22 µm membrane filter (Whatman). 

High-performance liquid chromatography (HPLC) was used to determine the protopanaxadiol (PPD) and protopanaxatriol (PPT) ginsenoside compositions of the extracts, following Suarez et al. [29]. The centrifuged extracts (10 μL) were injected onto a ZORBOX Eclipse Plus C8 column (2.1 × 50 mm, 1 μm, Agilent Technologies, Santa Clara, CA, USA) and eluted with a gradient of solvent B (90% acetonitrile, 0.1% formic acid, and 1 mg/L sodium acetate) in solvent A (0.1% formic acid and 1 mg/L sodium acetate). Starting conditions were 25% solvent B in 75% solvent A for 1 min, followed by a linear gradient to 35% solvent B over 2 min, then, 95% solvent B over 6 min, and then maintained at 95% solvent B for 1 min before returning to the starting conditions. The flow rate was 0.4 mL/min, and the eluent was monitored at 203 nm before infusion into an Agilent 6320 TOF (Agilent, Mississauga, ON, Canada mass spectrometer through a dual-spray electrospray ionization (ESI) source with gas temperature of 325 °C flowing at 12 L/min and a nebulizer pressure of 45 psi. The fragmentor voltage was set to 120 V with a Vcap of 4500 V. Automated internal calibration was carried out using reference ions 121.0508 and 922.0096. The column was conditioned with 25% solvent B for 9 min between samples and maintained at 40 °C. Ginsenosides were detected as their Na^+^ adducts in positive ion mode (M + Na^+^H).

### 2.8. Detached Root Assay 

*Ilyonectria mors-panacis* isolate IMP.ND4Z15 was cultured on V8 agar (200 mL/l V-8 juice, 3.0 g/L CaCO_3_, 15.0 g/L agar, pH 7.2) for 4 weeks in the dark at 22 °C. Macroconidia were harvested by adding 5 mL sterile distilled H_2_O to the plates and the spore solution was adjusted to 1 × 10^6^ spores/mL, based on hemocytometer counts. Roots of *P. qiunquefolius* were surface sterilized with 75% ethanol for 10 min followed by 5% bleach for 5 min, and then thoroughly washed with sterile distilled H_2_O. Wounds (approximately 1.5 mm wide and 9 mm deep) were created on the roots with a sterilized needle, and 15 µL of either water (control), LL broth extract, or LLG broth extract were placed into the cavities. After 2 h, 15 µL spore suspension was added to each cavity, and the roots were incubated on sterile filter paper saturated with sterile distilled H_2_O in sterile Petri dishes at 22 ± 2 °C. Control roots (negative control) were wounded and mock inoculated with only sterile distilled H_2_O. Lesions were recorded at 12 dpi by tracing the lesion area on acetate sheets and scanning the sheets to make a pdf file, and the traced areas were quantified using ImageJ software version 1.22 (https://imagej.net). 

### 2.9. Statistical Analysis 

Data were compared through analysis of variance (ANOVA) using Minitab version 16, and when significant treatment effects were found, means were compared using Fisher’s LSD Test accessed 4 December 2018 with a level of significance at *p* = 0.05.

## 3. Results

### 3.1. Identification and Phylogenetic Relationships of Pseudomonas plecoglossicida Isolates 

Thirteen colony morphotypes were observed for bacteria isolated from soil previously planted with *P. quinquefolius* (Table 2). Among these, only roots treated with cell-free extracts of LLG broth incubated with isolates 17-08R and 17-16R showed significantly increased lesion size produced by *I. mors-panacis* isolate IMP.ND4Z15 compared with the control non-inoculated LLG broth, indicating immunosuppression activity by these two isolates. Isolate 17-08R was selected because the lesion size showed the greatest difference from the control. 

Isolate 17-08R was identified as *Pseudomonas plecoglossicida*, based on its 16S rRNA sequence most closely matching those of *P. plecoglossicida* isolate XSDHY-P (CP031146.1), *P. plecoglossicida* isolate XSDHY-P (CP010359.1), and *Pseudomonas monteillii* isolate SB 3067 (GU191931.1), all with e-values of 0.0. The genome sequence of *P. plecoglossicida* isolate 17-08R was obtained from 11,961,314 100 bp raw read sequences, which were assembled with SOAP assembler, giving an N50 value of 247 kb, 261 contigs, and an assembled genome size of 5 Mb. 

**Figure 1:**
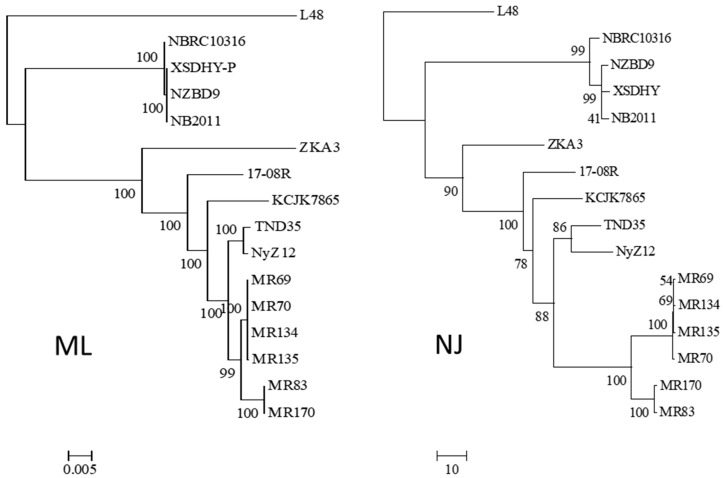
Relatedness of *P. plecoglossicida* isolates listed in Table 1. Rooted maximum likelihood dendrogram (**left**) based on the concatenated nt sequences of 16S rRNA, *rpoB*, *rpoD*, and *gyrB* genes previously used to show the relationship of *Pseudomonas putida* group by Mulet et al. [22], and a neighbor-joining dendrogram (**right**) based on a similarity matrix of homologous genes predicted using AUGUSTUS. The 16S rRNA, *rpoB*, *rpoD* and *gyrB* sequences were aligned using MUSCLE for the ML tree, and the similarity matrix for the NJ tree was obtained using a reciprocal best hit BLASTN. The ML dendrogram was created using RAxML and the NJ tree was generated using the R package APE. Both trees included *Pseudomonas entomophila* L48 as the outgroup, 1000 bootstrap replications were run, and results were viewed using MEGA6. The ML scale bar represents an estimate of the number of nucleotide substitutions per site, and the NJ scale bar represents percent nucleotide sequence.

Using the 16S rRNA sequence of isolate 17-08R as a query, genome sequences of 14 other isolates of *P. plecoglossicida* were collected from NCBI, as well as that of a *Pseudomonas entomophila* isolate as an outgroup. All the *P. plecoglossicida* isolates were from fish or soil. An ML phylogenetic tree based on the concatenated nt sequences of 16S rRNA, *rpoB*, *rpoD*, and *gyrB* showed that the four isolates from fish tissues were distinct from the eleven isolates from soil, including 17-08R (Figure 1). The soil isolates showed one subcluster with 100% bootstrap support containing isolates TND35 and NyZ12, a nicotine-degrading bacterium from India and a cyclohexylamine-degrading bacterium from China. There was another subcluster with 99% bootstrap support containing isolates MR69, MR70, MR134, and MR135 from polluted wetland soil from Nigeria, and a third subcluster with 100% bootstrap support containing isolates MR83 and MR170, also from polluted wetland soil from Nigeria. Isolates 17-08R, ZKA3, and KCJK7865 were the most distantly related of the soil isolates. An NJ phylogenetic tree for the same isolates using AUGUSTUS predicted total genome nt sequences showed a highly similar structure (Figure 1).

### 3.2. Genomic Analysis of P. plecoglossicida Isolates

**Figure 2:**
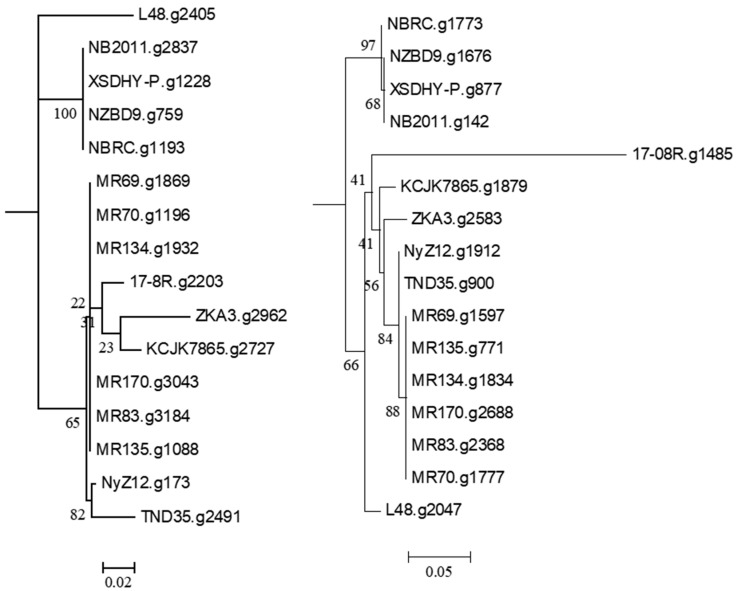
Relatedness of predicted proteins in the genomes of *P. plecoglossicida* isolates listed in Table 1 queried with glycoside hydrolase proteins with described functions of ginsenoside transformation, metabolism, catalysis, and/or hydrolysis. Protein sequences of homologs of 17-08R.g2203 (PpBGLU) (**left**) and 17-08R.g1485 (PpBHEX) (**right**) were aligned using MUSCLE, and rooted maximum-likelihood dendrograms were created using RAxML with *Pseudomonas entomophila* L48 as an outgroup and 1000 bootstrap replicates and were viewed via MEGA6. Scale bars indicate the distance of the sequences from one another based on the average expected differences in the protein sequences per site.

Searching the *P. plecoglossicida* 17-08R genome using predicted bacterial protein sequences from NCBI with described functions of ginsenoside transformation, metabolism, catalysis, and/or hydrolysis resulted in the identification of the predicted protein sequences 17-08R.g2203 (e-value 1.23 × 10^−142^) and 17-8R.g1485 (e-value 8.55 × 10^−23^). 17-08R.g2203 was predicted to be a β-glucosidase in the glycoside hydrolase 3 family and was designated PpBGLU. It was not predicted to be secreted through either a classical or non-classical secretion pathway, but it contained a periplasmic domain. A neighbor-joining tree of the single homolog of 17-08R.g2203 identified in the genome of each of the selected *P. plecoglossicida* isolates showed that those isolated from either fish or soils were in separate clusters, with 17-08R.g2203 clustering with other soil isolates (Figure 2 left), while 17-8R.g1485 was predicted to be a β-N-acetylhexosaminidase belonging to the glycoside hydrolase 3 family and was designated PpBHEX. It was also not predicted to be secreted, nor did it contain a periplasmic domain. A neighbor-joining tree of the single homolog found in each of the selected *P. plecoglossicida* and *P. entomophila* isolates showed the fish and soil isolate sequences clustered separately, with 17-08R.g1485 being the most basal among the soil isolate sequences (Figure 2 right). Using queries from NCBI, no homologs of other glycoside hydrolases, β-d-xylosidase, alpha-L-rhamnosidase, or alpha-L-arabinofuranosidase were found among the predicted proteins of the *P. plecoglossicida* 17-08R (cut-off e-value of 1 × 10^−5^). 

**Figure 3:**
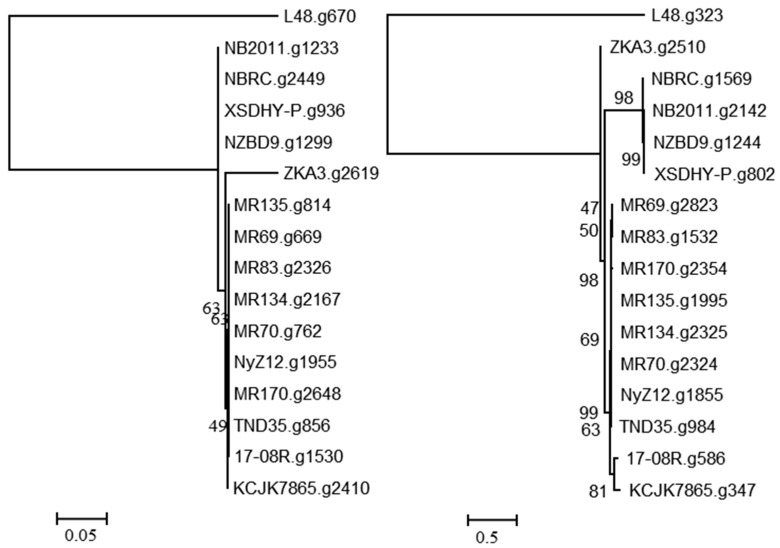
Relatedness of predicted proteins in the genomes of *P. plecoglossicida* isolates listed in Table 1 queried with outer membrane proteins with previously described functions of ginsenoside hydrolysis. Protein sequences of homologs of 17-08R.g1530 (PpOmpA/MotB) (**left**) and 17-08R.g586 (PpTonB) (**right**) were aligned using MUSCLE, and rooted maximum likelihood dendrograms were created using RAxML with 1000 bootstrap replicates and viewed using MEGA6. Scale bars indicate the distance of the sequences from one another based on the average expected differences in the protein sequences per site. Homologs from *Pseudomonas entomophila* L48 were used as an outgroup.

A search using the predicted bacterial proteins from NCBI with described functions of ginsenoside hydrolysis identified 17-08R.g1530 (e-value 3.00 × 10^−30^) and 17-08R.g586 (e-value 4.00 × 10^−5^) in the *P. plecoglossicida* 17-08R genome. A single homolog of 17-08R.g1530 was found in each genome of the *P. plecoglossicida* isolates, with the predicted proteins of the fish and soil isolates clustering separately and the 17-08R.g1530 sequence among those of other soil isolates (Figure 3 left). The 17-08R.g1530 sequence was predicted to be an outer membrane protein A (OmpA)/MotB family protein and was designated PpOmpA/MotB. Sequence 17-08R.g586 also had a single homolog in each of the *P. plecoglossicida* isolates, with one from soil, ZKA3.g2510, being the most distinctive, and separate clusters for all the remaining soil isolates and fish isolate sequences (Figure 3 right). The predicted protein for 17-08R.g586 was a TonB-dependent receptor outer membrane protein and was designated PpTonB. 

**Figure 4:**
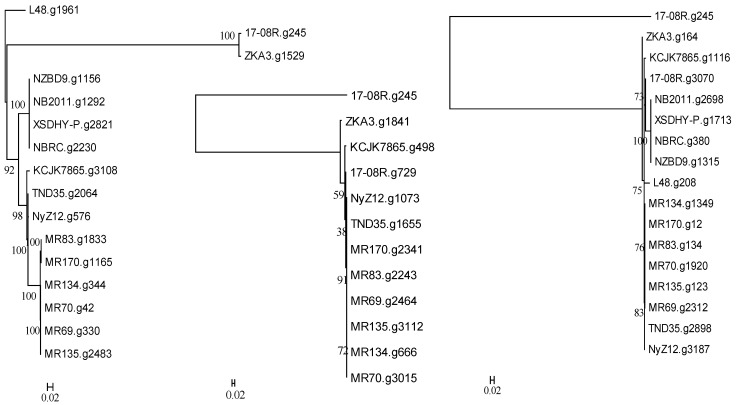
Relatedness of predicted proteins in the genomes of *P. plecoglossicida* isolates listed in Table 1 queried with glycoside oxidoreductase proteins having previously described functions of deglycosylation of ginsenosides. Protein sequences of homologs of 17-08R.g245 (PpGO1) (**left**), 17-08R.g729 (PpGO2) (**middle**), and 17-08R.g3070 (PpGO3) (**right**) were aligned using MUSCLE, and rooted maximum-likelihood dendrograms were created using RAxML with 1000 bootstrap replications and viewed by MEGA6. Scale bars indicate the distance of the sequences from one another based on the average expected differences in the protein sequences per site. *Pseudomonas entomophila* L48 or 17-08R.g245 were used as an outgroup.

A search of the genome of *P. plecoglossida* 17-08R with a predicted bacterial protein at NCBI with a described function of deglycosylation of ginsenosides and related to a TAT-pathway signal protein did not identify a homolog (e-value 1.00 × 10^−5^ cut-off). However, searching using a predicted bacterial protein at NCBI with a described function of deglycosylation of ginsenosides resulted in the identification of 17-08R.g245 (e-value 1.18 × 10^−44^), 17-08R.g729 (e-value 1.63 × 10^−27^), and 17-08R.g3070 (e-value 3.07 × 10^−6^). They were all predicted to be glycoside oxidoreductases, with only 17-08R.g729 predicted to be secreted, through a non-classical pathway. A single homolog of 17-08R.g245 (designated PpGO1) was found in each genome of the selected *P. plecoglossicida* isolates, with fish and soil isolate sequences clustering separately but with ZKA3.g1529 and 17-08R.g245 in a separate more distant cluster (Figure 4 left). A single homolog of 17-08R.g729 (designated PpGO2) was found in each of the genomes of the soil isolate, but none were found in the fish isolates or *P. entomophila* L48. A tree showed that 17-08R.g729 was highly similar to the sequences of the other soil isolates but was quite distinct from PpGO1 (17-08R.g245) (Figure 4 middle). A tree of the single homolog of 17-08R.g3070 (designated PpGO3) found in each selected *P. plecoglossicida* genome revealed that the fish isolate sequences clustered separately, but some soil isolate sequences, including 17-08R.g3070, did not cluster with the majority of the soil isolate sequences (Figure 4 right).

### 3.3. Pseudomonas Plecoglossicida Growth with Ginseng Root Extract 

**Figure 5:**
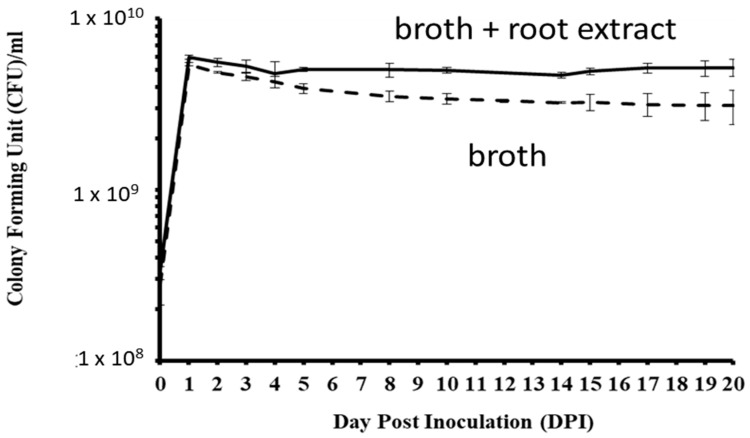
Growth of *P. plecoglossicida* 17-08R in LL broth (dashed line) or LLG (LL broth + 48 mg/mL *P. quinquefolius* root extract) (solid line). Values are an average of 3 replications with standard deviation bars shown.

In LL media, CFUs of *P. plecoglossicida* 17-08R significantly increased from 0 to 1 dpi and significantly decreased by 5 dpi, and there were no significant changes between 5 and 20 dpi (Figure 5). In LLG media that contained *P. quinquefolius* root extract, CFUs significantly increased from 0 to 1 dpi and then did not significantly change between 2 and 20 dpi. The CFUs of *P. plecoglossicida* 17-08R were significantly higher in LLG media than LL media at 1, 2, 4, 5, 8, 10, 14, 15, 17, 19, and 20 dpi.

### 3.4. Changes in Ginsenosides during P. plecoglossicida Growth with Ginseng Root Extract

**Figure 6:**
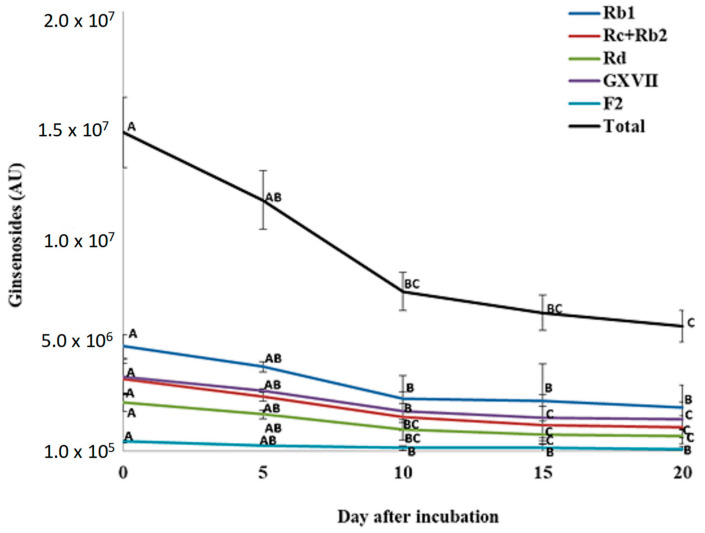
Total and individual protopanaxadiol ginsenoside (PPD) levels over time during incubation of *Panax quinquefolius* root extract in LLG broth with *P. plecoglossicida* 17-08R. Values of AU (arbitrary unit) for each ginsenoside are the mean of three replications compared using Fisher’s LSD at *p* = 0.05. Points with letters in common for each individual ginsenoside are not significantly different.

**Figure 7:**
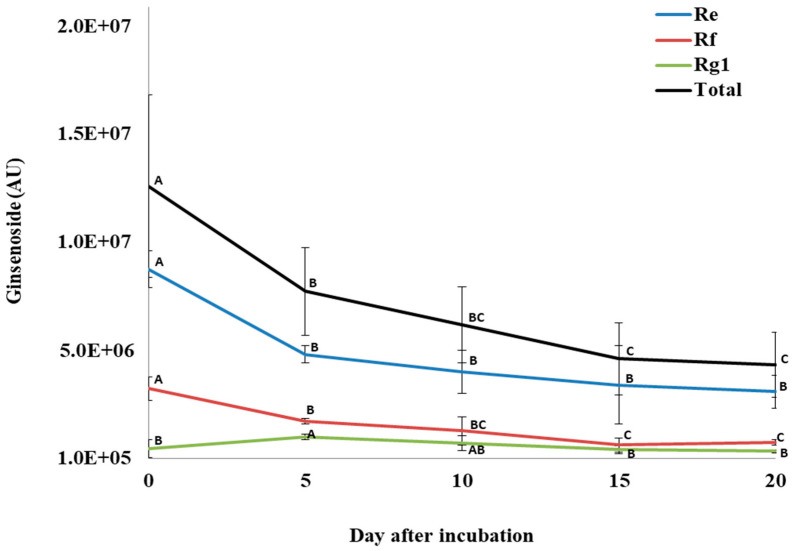
Total and individual protopanaxatriol ginsenoside (PPT) levels over time during incubation of *Panax quinquefolius* root extract in LLG broth with *P. plecoglossicida* 17-08R. Values of AU (arbitrary unit) for each ginsenoside are the mean of 3 replications compared using Fisher’s LSD method at significance of *p* = 0.05. Points with letters in common for each individual ginsenoside are not significantly different.

For total PPD ginsenosides, incubation of *P. quinquefolius* crude root extract with *P. plecoglossicida* 17-08R resulted in a significant decline from 1.49 × 10^7^ AU at 0 dpi to 5.91 × 10^6^ AU at 20 dpi, which was a 60.4% reduction (Figure 6). Among the individual PPDs detected, none significantly decreased between 0 and 5 dpi. However, all significantly decreased between 0 and 10 dpi, but only GXVII ginsenoside significantly decreased between 10 and 20 dpi. Regarding total PPT ginsenosides, incubation of *P. quinquefolius* crude root extract with *P. plecoglossicida* 17-08R showed a non-significant drop from 1.27 × 10^7^ AU at 0 dpi to 4.43 × 10^6^ AU at 20 dpi, which was a 67.1% reduction (Figure 7). Among the individual PPTs detected, both Re and Rf ginsenosides significantly decreased between 0 and 5 dpi, and Rg1 ginsenoside significantly increased between 0 and 5 dpi. Only Rf and Rg1 significantly decreased between 5 and 20 dpi.

### 3.5. Effect of Ginseng Root Extract Incubated with P. plecoglossicida on Root Rot Disease

Lesion sizes caused by *I. mors-panacis* IMP.ND4Z15 on *P. quinquefolius* roots treated with sterile non-inoculated LL or LLG broth media were not significantly different from each other, indicating that the inclusion of ginseng root extract into culture media without *P. plecoglossicida* did not affect the infection (Table 3). Roots treated with 20 dpi *P. plecoglossicida*-cell-free extracts from LL broth showed root rot lesion sizes that were also not significantly different from treatment with non-inoculated LL and LLG broths. However, root rot lesion sizes were significantly larger compared with the other treatments with 20 dpi *P. plecoglossicida*-cell-free extracts from LLG broth.

## 4. Discussion

Athough several species of *Pseudomonas* have been isolated from ginseng soil, such as *P. thivervalensis* [30], *P. putida* [31], and *P. fluorescens* [32], this is the first report of the isolation of *P. plecoglossicida* from ginseng soil. A reclassification of the *P. putida* group by Mulet et al. [22], based on combined 16S rRNA, *gyrB*, *rpoB*, and *rpoD* sequences, divided it into *P. cremoricolorata*, *P. fulva*, *P. mosselii*, *P. monteilii*, *P. parafulva*, *P. plecoglossicida*, and *P. putida.* However, based on 16S rRNA sequencing alone, the species are hard to distinguish, with *P. plecoglossicida* being 98.6% to 99.6% similar to *P. putida* [33]. Thus, it could be that an earlier report of *P. putida* isolated from soil of cultivated *P. ginseng* in Korea was actually *P. plecoglossicida*, as only 16S rRNA sequencing was performed [34]. However, this would clearly be the first report of *P. plecoglossicida* in *P. quinquefolius* soil in North America. 

*Pseudomonas plecoglossicida* has previously been isolated from various fish tissues worldwide, including those of *Larimichthys crocea* [35,36] and *Plecoglossus altivelis* [33,37], and from soil, including soil contaminated with cyclohexylamine and nicotine [38,39], peat marsh soil with lignin- and cellulose-degrading activity [40], wetland soil contaminated with sewage [41], and a non-described soil [42]. Thus, it was not entirely unexpected to have isolated *P. plecoglossicida* from ginseng soil in Canada.

Comparing the genomes of several isolates of *P. plecoglossicida*, using either concatenated 16S rRNA, *gyrB*, *rpoB*, and *rpoD* genes as per Mulet et al. [22], or using DNA sequences for total predicted proteins, showed that *P. plecoglossicida* 17-08R was similar to other soil isolates. However, all the soil isolates were distinct from the fish isolates, which clustered together. Similarly, Adelowo et al. [41] made a phylogenetic tree using 520 single-copy core gene sequences per strain and showed that *P. plecoglossicida* isolates from fish tissues were also highly distinct from those from soil, while *P. entomophila* L48 from fruit flies was included as a related outgroup and did not cluster with any of the *P. plecoglossicida* isolates. Considering the results of Adelowo et al. [41] and this study, it is proposed that *P. plecoglossicida* isolates from fish and soil are non-overlapping and possibly different sub-species, but are unlikely to be different species because they have 99% to 100% nt identity in 16S rRNA genes.

As ginsenosides are secreted by ginseng roots [7] and can enhance the growth of soil bacteria that are able to utilize them as carbon sources [9], the sequenced genome of *P. plecoglossicida* 17-08R was examined for the presence of predicted proteins potentially involved in ginsenoside transformation according to other reports. The genome of *P. plecoglossicida* 17-08R contained genes for glycoside hydrolases (β-glucosidase and β-N-acetylhexosaminidase), outer membrane proteins (OmpA/MotB and TonB-dependent receptor), and glycoside oxidoreductase, which are all related to genes described as functioning in ginsenoside modification in other bacterial species. β-glucosidase can remove glucose from C3 or C20 of ginsenosides [12]. The periplasmic domain of the β-glucosidase from *P. plecoglossicida* 17-08R indicated that it was located between the inner and outer membrane along with other periplasmic enzymes, such as alkaline phosphatase, cyclic phosphodiesterase, acid phosphatase, and 5′-nucleotidase [43]. While another gene for a glycoside hydrolase, β-N-acetylhexosaminidase, was found in the genome, which should be able to hydrolyze the terminal non-reducing N-acetyl-d-hexosamine residues [44], ginsenosides have no N-acetyl-d-hexosamine, and this glycoside hydrolase would thus be unlikely to be involved in deglycosylation of ginsenosides. The absence of detectable genes in the genome for β-d-xylosidase, rhamnosidase, and arabinosidase indicated that removal of those sugars from ginsenosides would not be possible via glycoside hydrolases of *P. plecoglossicida* 17-08R. The OmpA/MotB and TonB-dependent receptor proteins in *P. plecoglossicida* 17-08R matched those from *Sphingobacterium multivorum* GIN723 that are involved in ginsenoside deglycosylation, based on the purification of two proteins showing enzymatic activity for deglycosylation of compound K to PPD(S) and F1 to PPT(S) [45]. However, OmpA/MotB is an outer membrane channel protein associated with the type VI secretion system [46], and the TonB-dependent receptor is associated within protein complexes for transporting solutes and macromolecules [47]. Thus, these proteins may not be directly involved in ginsenoside transformation but could allow ginsenosides to enter into the periplasm where other enzymes can act on them, such as possibly the periplasmic β-glucosidase of *P. plecoglossicida* 17-08R. The three glycoside oxidoreductases predicted from the genome of *P. plecoglossicida* 17-08R were similar to those from *Rhizobium* sp. associated with cleavage of the glucose at the C-20 position of compound K converting it into PPD(S) [13]. Among the three glycoside hydrolases, only PpGO-2 might be secreted. Thus, it could be transforming ginsenosides in the media, but the activity by the other two glycoside hydrolases may occur in the cytoplasm after ginsenoside absorption, if they actually act on ginsenosides.

As *Pseudomonas plecoglossicida* 17-08R showed higher populations in LLG broth than LL broth, it may have been using components of the ginseng root extract as nutrients. One possibility is that the bacteria were utilizing soluble nutrients from ginseng roots, such as amino acids, proteins, and carbohydrates [48]. However, ginsenosides are a major component of ginseng root, comprising 3–8% of its dry weight [49], and they contain glucose, arabinose, xylose, and rhamnose attached to the core dammarane structure [50]. Thus, a second possibility is that sugars on ginsenosides were cleaved and used by *P. plecoglossicida* 17-08R as nutrients, such as has been reported for other ginseng soil bacteria [10]. 

Analysis of ginsenosides in cell-free LLG broth supernatant showed that total PPD and PPT ginsenosides decreased over time in cultures of *P. plecoglossicida* 17-08R. All the detected individual PPD ginsenosides decreased over time in a similar pattern, indicating no specificity based on the specific PPD ginsenoside. However, while the PPT ginsenosides Rf and Re declined similarly to total PPT ginsenosides over time, Rg1 increased initially. As Rf has two glucoses at C6, Re has two glucoses at C6 and one glucose at C20, and Rg1 has one glucose at C6 and one glucose at C20, Re could be converted to Rg1 by removing a sugar from C6, but if the same had occurred with Rf, then Rg2 would be created, which was not detected. Other than the significant increase in Rg1 between 0 and 5 dpi, all the other PPD and PPT ginsenosides progressively decreased from day 0 to day 20 of incubation with *P. plecoglossicida* 17-08R. 

Based on the genes with potential for ginsenoside transformation by *P. plecoglossicida* 17-08R, a theoretical conversion process can be proposed. Following uptake by OmpA/MotB and/or TonB-dependent receptor, the periplasmic β-glucosidase would remove glucose from certain ginsenosides, eventually creating partially deglycosylated forms, such as compound K or gypenoside LXXV [51,52]. However, other ginsenosides would be less affected as they also contain arabinofuranosyl, arabinopyranosyl, and/or rhamnopyranosyl, and no genes for enzymes acting on those sugars were found in *P. plecoglossicida* 17-08R. However, one or more of the predicted cytoplasmic or secreted glycoside oxidoreductases could remove most or all of the remaining sugars to create aglycone PPD and PPT ginsenosides. The glycoside oxidoreductase of *Rhizobium* sp. strain GIN611 reacted with the partially deglycosylated ginsenoside compound K, removing the last glucose at C20 to create aglycone PPD(S) [13]. It could also oxidize sugars of other ginsenosides, including the PPD ginsenosides, Rb1, Rb2, Rb3, Rc, F2, and Rh2, the PPT ginsenosides Re and F1, and even an isoflavone, daidzin, and it showed a broad range of specificity to hydrolysing sugar moieties, such as glucose, galactose, and xylose. However, no highly deglycosylated ginsenosides were detected in the media during the growth of *P. plecoglossicida* 17-08R. This was not likely to have been due to the type of HPLC analysis, as less polar deglycosylated forms of ginsenosides, including aglycone PPD(S) and PPT(S), should be retained in the C8 column and eluted using the solvent gradients described in this study, thus being detectable. Therefore, it remains unclear how ginsenosides are transformed by *P. plecoglossicida* 17-08R.

In addition to ginseng root extract increasing the population of *P. plecoglossicida* 17-08R in broth, the formation of a cell-free extract at 20 dpi allowed *I. mors-panacis* to produce larger lesions on roots compared with an extract from bacteria growing in broth without ginsenosides. Thus, some material was created in the bacterial growth media containing ginseng root extract that enabled increased levels of fungal root rot, and this material was not present in ginseng roots prior to exposure to the bacterium, as non-inoculated media containing ginseng root extract did not have that effect. The increase in lesion size with *I. mors-panacis* was similar to that in roots treated with an extract from soil previously planted with ginseng, compared with lesion sizes of roots treated with an extract of ginseng roots or soil not previously used for ginseng production [14]. Lesion size appeared to be largely due to the suppression of jasmonate-related gene expression following infection, implying that the compound(s) suppressed the innate immune system of ginseng during *I. mors-panacis* infection. 

Certain ginsenosides in soil are allelopathic to ginseng, such as Rg1 and Rb that inhibited seedling emergence and growth and root cell vigour in *P. notoginseng* [53], and Rb1, Rb2, and Rd that reduced seedling height and radicle length in *P. quinquefolius* [54]. Allelopathic compounds can enter soil through various means including root exudates and tissue degradation. For ginseng, considerable amounts of root debris remain after mechanical harvesting, which decay in the soil quickly over time, affecting soil microbial populations and releasing compounds, including ginsenosides [29]. Allelopathy can be mediated by soil microbes by transforming allelochemicals in soil into more toxic products or converting non-toxic compounds into allelochemicals, thereby enhancing allelopathy [55]. In addition, allelopathy may be related to plant disease resistance, such as through root exudates altering innate immune gene expression [56]. An example is allantoin secreted by rice roots, which can be transformed in the soil by bacteria [57]; allantoin can induce the expression of plant JA-responsive genes, affecting the susceptibility of *A. thaliana* to bacterial pathogens [58]. Thus, it is not unreasonable to postulate that compounds released from ginseng roots decaying after commercial harvesting could be converted by soil microbes, such as *P. plecoglossicida* 17-08R, into compounds that modulate the innate immune system of ginseng, possibly by affecting jasmonate signaling. This could then suppress the induction of jasmonate-related defenses triggered during *I. mors-panacis* infection, resulting in more root rot, which could be a contributing factor to ginseng replant disease. Thus, the proposed host-related factor in soil following ginseng harvest that contributes to ginseng replant disease [1] could be microbially transformed ginseng root debris compounds.

## 5. Conclusions

A bacterium isolated from soil previously planted with ginseng was able to grow in broth with ginseng root extract, creating compound(s) that affected *P. quinquefolius* roots so that the lesion sizes caused by *I. mors-panacis* were increased, similar to the effect reported by Behdarvandi and Goodwin [14] with an extract from soil previously planted with ginseng. Ginseng root extract alone did not demonstrate this activity. While ginsenosides may have been involved, there was no evidence for novel ginsenosides or significantly increased deglycosylated ginsenosides in the media extract that increased root rot, despite the potential for the production of deglycosylated ginsenosides based on the predicted proteins of the genome of *P. plecoglossicida* 17-08R. Thus, the nature of the compound(s) remains to be determined. However, the results indicated that a possible explanation for the appearance of compound(s) able to suppress resistance of ginseng to root rot in soil previously planted with ginseng may the conversion by various ginseng soil microbes of compounds present in ginseng roots. 

## Figures and Tables

**Table 1:** Genomes of *P. plecoglossicida* collected from the NCBI nr database based on high-nt matches using the 16S DNA sequence of isolate 17-08R as a query. *Pseudomonas entomophila* (L48) was included as an outgroup as it was previously used as an outgroup in a comparison of *Pseudomonas putida* genomes, which was the previous species classification of *P. plecoglossicida* [21].

Organism	Isolate	Assembly Accession	Location	Source	Source Description
*P. plecoglossicida*	NZBD9	GCA_002814195.1	Ningde/China	fish	*Larimichthys crocea*
*P. plecoglossicida*	XSDHY-P	GCA_003391255.1	Ningbo/China	fish	*Larimichthys polyactis*
*P. plecoglossicida*	NB2011	GCA_000412715.1	East coast/China	fish	*Larimichthys polyactis*
*P. plecoglossicida*	NBRC10316	GCA_000730665.1	Tokushima Pref./Japan	fish	*Plecoglossus altivelis*
*P. plecoglossicida*	NyZ12	GCA_000831585.1	Wuhan/China	soil	Cyclohexylamine-degrading bacterium
*P. plecoglossicida*	KCJK7865	GCA_003062165.1	Florida/USA	soil	Not determined
*P. plecoglossicida*	ZKA3	GCA_003633555.1	Zakynthos/Greece	soil	Biomass-degrading bacterium
*P. plecoglossicida*	TND35	GCA_000764405.1	Tamil Nadu/India	soil	Nicotine-degrading bacterium
*P. plecoglossicida*	MR69	GCA_002864775.1	Ibadan/Nigeria	soil	Polluted wetlands
*P. plecoglossicida*	MR135	GCA_002864795.1	Ibadan/Nigeria	soil	Polluted wetlands
*P. plecoglossicida*	MR170	GCA_002864845.1	Ibadan/Nigeria	soil	Polluted wetlands
*P. plecoglossicida*	MR83	GCA_002864865.1	Ibadan/Nigeria	soil	Polluted wetlands
*P. plecoglossicida*	MR134	GCA_002864885.1	Ibadan/Nigeria	soil	Polluted wetlands
*P. plecoglossicida*	MR70	GCA_002864905.1	Ibadan/Nigeria	soil	Polluted wetlands
*P. plecoglossicida*	17-08R	PRJNA1153425	Simcoe/Canada	soil	3-year post-harvest ginseng soil
*P. entomophila*	L48	GCA_000026105.1	Western Guadeloupe	fruit fly	*Drosophila melanogaster*

**Table 2:** Description of *P. quinquefolius* soil bacterial isolates. Colony size, color, and shape determined after 48 h growth on LB at 30 °C. Lesion size caused by *I. mors-panacis* isolate IMP.ND4Z15 on *P. quinquefolius* roots treated 2 h prior to inoculation with cell-free extract of 20-day-old cultures of each isolate grown in LLG. Root lesion size measured at 12 dpi. Control was cell-free extract of LLG not inoculated with bacteria. Means are the average of one experiment with 3 to 5 lesions, and means followed by a letter in common are not significantly different according to the Fisher’s LSD test (*p* = 0.05).

Isolate	Soil Year after Harvest	Colony Size ^a^	Colony Color ^b^	Lesion Size (cm^2^)
17-06R	3	very small	creamy to colorless	0.15 E
17-07R	3	small	creamy	0.19 CDE
17-08R	3	small	creamy to yellow	0.27 A
17-09R	3	small	orange	0.16 CDE
17-10R	25	large	light brown to gray	0.21 BCD
17-11R	25	very large	whitish	0.22 ABC
17-12R	5	large	creamy	0.19 CDE
17-13R	1	small	white	0.18 CDE
17-14R	0.08	very small	white	0.16 DE
17-15R	0.08	large	white	0.16 E
17-16R	1	large	creamy	0.25 AB
17-17R	10	large	white–creamy centre	0.19 CDE
17-18R	10	large	orange	0.19 CDE
Control	-	-	-	0.18 CDE

^a^ Colony sizes defined as very small < 1.0 mm, small ~ 1.0 mm, large between 1.0 and 3.0 mm, very large > 3.0 mm diameter at 48 h on LB at 30 °C. ^b^ Color determined at 48 h on LB at 30 °C.

**Table 3:** Effect of cell-free broth extracts of LL or LLG inoculated or not inoculated with *P. plecoglossicida* 17-08R on the size of lesions caused by *I. mors-panacis*. For *P. plecoglossicida* 17-08R, bacteria were grown in broth for 20 days at 30 °C. Broths were evaporated to dryness under vacuum at 40 °C and dissolved in 1 mL of dsH2O. Then, 15 μL of the extract was added to wounded surface sterilized roots of *P. quinquefolius* 2 h before application of 15 μL of 10^6^ spores/mL of *I. mors-panacis* isolate IMP.ND4Z15. After 12 days at 22 °C, lesion size was quantified with ImageJ software version 1.22.Means are from three replications with six lesions measured per replication and compared using Fisher’s LSD test (*p* = 0.05). Means with letters in common are not significantly different.

Media	*P. plecoglossicida* 17-08R	Lesion Size (cm^2^)
LL broth	−	0.16 B
LLG broth	−	0.18 B
LL broth	+	0.17 B
LLG broth	+	0.23 A

## Data Availability

The data that support the findings of this study are available on request from the corresponding author (P.H.G) (email address: pgoodwin@uoguelph.ca). The genome of the *P. plecoglossicida* isolate in this study is available through NCBI under BioProject TBD.

## Supplementary Materials

The following supporting information can be downloaded at: https://www.mdpi.com/article/10.3390/biology13090671/s1, Table S1: Bacterial glycoside hydrolase protein sequences obtained from NCBI using the terms “ginsenoside transformation”, “metabolism of ginsenoside”, and “transform ginsenoside” as queries; Table S2. Bacterial glycoside hydrolase protein sequences obtained from NCBI for xylosidase using the term ‘ginsenoside hydrolyzing’, rhamnosidase using the term ‘(pseudomonas [Organism]) AND rhamnosidase)*’*, and α-L-arabinofuranosidase using the term ‘(pseudomonas[Organism]) AND α-L-arabinofuranosidase ‘ as queries; Table S3. Bacterial outer membrane protein sequences obtained from NCBI using the term ‘ginsenoside aglycons’ as a query; Table S4. Bacterial glycoside oxidoreductase and TAT-pathway signal protein sequences obtained from NCBI using the term ‘deglycosylation of ginsenosides’ as a query.

## References

[1] Westerveld S.M., Shi F. (2021). The history, etiology, and management of ginseng replant disease: A Canadian perspective in review. Can. J. Plant Sci..

[2] Goodwin P.H. (2022). The rhizosphere microbiome of ginseng. Microorganisms.

[3] Nguyen N., Kim Y., Hoang V., Subramaniyam S., Kang J., Kang C., Yang D. (2016). Bacterial diversity and community structure in Korean ginseng field soil are shifted by cultivation time. PLoS ONE.

[4] Dong L., Xu J., Li Y., Fang H., Niu W., Li X., Zhang Y., Ding W., Chen S. (2018). Manipulation of microbial community in the rhizosphere alleviates the replanting issues in *Panax ginseng*. Soil Biol. Biochem..

[5] Zhang J., Fan S., Qin J., Dai J., Zhao F., Gao L., Lian X., Shang W., Xu X., Hu X. (2020). Changes in the microbiome in the soil of an American ginseng continuous plantation. Front. Plant Sci..

[6] Brimecombe M.J., De Leij F.A., Lynch J.M. (2000). The effect of root exudates on rhizosphere microbial populations. The Rhizosphere.

[7] Nicol R.W., Yousef L., Traquair J.A., Bernards M.A. (2003). Ginsenosides stimulate the growth of soilborne pathogens of American ginseng. Phytochemistry.

[8] Taira S., Ikeda R., Yokota N., Osaka I., Sakamoto M., Mitsuro K., Sahashi Y. (2010). Mass spectrometric imaging of ginsenosides localization in *Panax ginseng* root. Am. J. Chin. Med..

[9] Miao X., Wang E., Zhou Y., Zhan Y., Yan N., Chen C., Li Q. (2023). Effect of ginsenosides on microbial community and enzyme activity in continuous cropping soil of ginseng. Front. Microbiol..

[10] Luo L.F., Yang L., Yan Z.X., Jiang B.B., Li S., Huang H.C., Liu Y.X., Zhu S.S., Yang M. (2020). Ginsenosides in root exudates of *Panax notoginseng* drive the change of soil microbiota through carbon source different utilization. Plant Soil..

[11] Eom S.J., Kim K.T., Paik H.K. (2018). Microbial bioconversion of ginsenosides in *Panax ginseng* and their improved bioactivities. Food Rev. Int..

[12] Wang L., An D.S., Kim S.G., Jin F.X., Lee S.T., Im W.T. (2011). *Rhodanobacter panaciterrae* sp. nov., a bacterium with ginsenoside-converting activity isolated from soil of a ginseng field. Int. J. Syst. Evol. Microbiol..

[13] Kim E.M., Kim J., Seo J.H., Park J.S., Kim D.H., Kim B.G. (2012). Identification and characterization of the *Rhizobium* sp. strain GIN611 glycoside oxidoreductase resulting in the deglycosylation of ginsenosides. Appl. Environ. Microbiol..

[14] Behdarvandi B., Goodwin P.H. (2023). Effect of soil and root extracts on the innate immune response of American ginseng (*Panax quinquefolius*) to root rot caused by *Ilyonectria mors-panacis*. Plants.

[15] Behdarvandi B., Hsiang T., Valliani M., Goodwin P.H. (2023). Differences in saprophytic growth, virulence, genomes, and secretomes of *Ilyonectria robusta* and *I. mors-panacis* isolates from roots of American ginseng (*Panax quinquefolius*). Horticulturae.

[16] Barba J.L.R., Maldonado A., Siaz R.J. (2005). Small-scale total DNA extraction from bacteria and yeast for PCR applications. Anal. Biochem..

[17] Lane D.J., Stackebrandt E., Goodfellow M. (1991). 16S/23S rRNA sequencing. Nucleic Acid Techniques in Bacterial Systematics.

[18] Kim M., Oh H.S., Park S.C., Chun J. (2014). Towards a taxonomic coherence between average nucleotide identity and 16S rRNA gene sequence similarity for species demarcation of prokaryotes. Int. J. Syst. Evol. Microbiol..

[19] Clarke L., Millar B.C., Moore J.E. (2003). Extraction of genomic DNA from *Pseudomonas aeruginosa*: A comparison of three methods. Br. J. Biomed. Sci..

[20] Camacho C., Coulouris G., Avagyan V., Ma N., Papadopoulos J., Bealer K. (2009). BLAST plus: Architecture and applications. BMC Bioinform..

[21] Wu X., Monchy S., Taghavi S., Zhu W., Ramos J., van der Lelie D. (2011). Comparative genomics and functional analysis of niche-specific adaptation in *Pseudomonas putida*. FEMS Microbiol. Rev..

[22] Mulet M., Gomila M., Lemaitra B., Lalucat J., Valdes E.G. (2012). Taxonomic characterisation of *Pseudomonas* strain L48 and formal proposal of *Pseudomonas entomophila* sp. nov. Syst. Appl. Microbiol..

[23] Madeira F., Park Y.M., Lee J., Buso N., Gur T., Madhusoodanan N., Basutkar P., Tivey A.R.N., Potter S.C., Finn R.D. (2019). The EMBL-EBI search and sequence analysis tools APIs in 2019. Nucleic Acids Res..

[24] Stamatakis A. (2014). RAxML version 8: A tool for phylogenetic analysis and post-analysis of large phylogenies. Bioinformatics.

[25] Tamura K., Stecher G., Peterson D., Filipski A., Kumar S. (2013). MEGA6: Molecular evolutionary genetics analysis version 6.0. Mol. Biol. Evol..

[26] Paradis E., Schliep K. (2018). ape 5.0: An environment for modern phylogenetics and evolutionary analyses in R. Bioinformatics.

[27] Dai J., Orsat V. (2010). Extraction of ginsenosides from American ginseng (*Panax quinquefolium* L.) root. Int. J. Food Eng..

[28] Cui L., Wu S.Q., Zhao C.A., Yin C.R. (2016). Microbial conversion of major ginsenosides in ginseng total saponins by *Platycodon grandiflorum* endophytes. J. Ginseng Res..

[29] Suárez I.D.S., Valliani M., Hsiang T., Goodwin P.H. (2023). Decay of root debris after harvesting American ginseng (*Panax quinquefolius*) and changes in soil chemistry and microbiology. Soil Syst..

[30] Liu T., Zhang J., Wang T., Li Z., Liang H., Jiang C., Tang H., Gao J., Jiang Y., Chen C. (2024). The novel *Pseudomonas thivervalensis* strain JI6 promotes growth and controls rusty root rot disease in *Panax ginseng*. Biol. Control..

[31] Shen L., Zhu G., Guo S., Li X., Xiao S., Xu J., Chen S. (2020). Isolation of a *Pseudomonas putida* strain that degrades p-hydroxybenzoic acid from the soil of a *Panax ginseng* field. Res. Sq..

[32] Park K.H., Lee C.Y., Son H.J. (2009). Mechanism of insoluble phosphate solubilization by *Pseudomonas fluorescens* RAF15 isolated from ginseng rhizosphere and its plant growth-promoting activities. Lett. Appl. Microbiol..

[33] Nishimori E., Kita-Tsukamoto K., Wakabayashi H. (2000). *Pseudomonas plecoglossicida* sp. nov., the causative agent of bacterial haemorrhagic ascites of ayu, *Plecoglossus altivelis*. Int. J. Syst. Evol. Microbiol..

[34] Hussein K.A., Joo J.H. (2015). Isolation and detection of genes responsible for pyoverdines biosynthesis in *Pseudomonas putida* KNUK9. Korean J. Soil Sci. Fert..

[35] Mao Z., Li M., Chen J. (2013). Draft genome sequence of *Pseudomonas plecoglossicida* strain NB2011, the causative agent of white nodules in large yellow croaker (*Larimichthys crocea*). Genome Announc..

[36] Tao Z., Wang G., Zhou S. (2018). Complete genome sequence of *Pseudomonas plecoglossicida* XSDHY-P, a strain that is pathogenic for the marine fish *Larimichthys crocea*. Microbiol. Resour. Announc..

[37] Kyrpides N., Huntemann M., Han J., Chen A., Mavromatis K., Markowitz V., Palaniappan K., Ivanova N., Schaumberg A., Pati A. (2014). Pseudomonas plecoglossicida NBRC 103162.

[38] Li X., Li C.Z., Mao L.Q., Yan D.Z., Zhou N.Y. (2015). Complete genome sequence of the cyclohexylamine-degrading *Pseudomonas plecoglossicida* NyZ12. Biotechnology.

[39] Raman G., Sakthivel N., Park S. (2015). Draft genome sequence of a novel nicotine-degrading bacterium, *Pseudomonas plecoglossicida* TND35. Genome Announc..

[40] Hatzinikolaou D. (2017). Genome Sequencing of Biomass-Degrading Isolates from Greek Habitats.

[41] Adelowo O., Vollmers J., Mäusezahl I., Kaster A., Müller J. (2018). Detection of the carbapenemase gene blaVIM-5 in members of the *Pseudomonas putida* group isolated from polluted Nigerian wetlands. Sci. Rep..

[42] Jeong K.C. (2018). Genome of Pseudomonas plecoglossicida KCJK7865.

[43] Beacham I.R. (1979). Periplasmic enzymes in gram-negative bacteria. Int. J. Biochem..

[44] Ichikawa T., Sugita T., Wang L., Yokoyama K., Nishimura K., Akemi Nishikawa A. (2004). Phenotypic switching and -N-acetylhexosaminidase activity of the pathogenic yeast *Trichosporon asahii*. Microbiol. Immunol..

[45] Kim E.M., Seo J.H., Kim J., Park J.S., Kim D.H., Kim B.G. (2013). Production of ginsenoside aglycons and Rb1 deglycosylation pathway profiling by HPLC and ESI-MS/MS using *Sphingobacterium multivorum* GIN723. Appl. Microbiol. Biotechnol..

[46] Sarris P.F., Zoumadakis C., Panopolous N.J., Scoulica E.V. (2011). Distribution of the putative type VI secretion system core genes in *Klebsiella* spp.. Infect. Genet. Evol..

[47] Martens E.C., Koropatkin N.M., Smith T.J., Gordon J.I. (2009). Complex glycan catabolism by the human gut microbiota: The bacteroidetes SUS-like paradigm. J. Biol. Chem..

[48] Sun H., Liu F., Sun L., Liu J., Wang M., Chen X., Xu X., Ma R., Feng K., Jiang R. (2016). Proteomic analysis of amino acid metabolism differences between wild and cultivated *Panax ginseng*. J. Ginseng Res..

[49] Court W.A., Reynolds L.B., Hendel J.G. (1996). Influence of root age on the concentration of ginsenosides of American ginseng (*Panax quinquefolium*). Can. J. Plant Sci..

[50] Shi Z.Y., Zeng J.Z., Wong A.S.T. (2019). Chemical structures and pharmacological profiles of ginseng saponins. Molecules.

[51] An D.S., Cui C.H., Lee H.G., Wang L., Kim S.C., Lee S.T., Jin F., Yu H., Chin Y.W., Lee H.K. (2010). Identification and characterization of a novel *Terrabacter ginsenosidimutans* sp. nov. beta-glucosidase that transforms ginsenoside Rb1 into the rare gypenosides XVII and LXXV. Appl. Environ. Microbiol..

[52] Kim D.W., Lee W.J., Gebru Y.A., Upadhyaya J., Ko S.R., Kim Y.H., Kim M.K. (2021). Production of minor ginsenosides CK and CY from naturally occurring major ginsenosides using crude β-glucosidase preparation from submerged culture of *Fomitella fraxinea*. Molecules.

[53] Yang M., Zhang X., Xu Y., Mei X., Jiang B., Liao J., Yin Z., Zheng J., Zhao Z., Fan L. (2015). Autotoxic ginsenosides in the rhizosphere contribute to the replant failure of *Panax notoginseng*. PLoS ONE.

[54] Jiao X.L., Bi X.B., Zhang X.S., Gao W.W. (2015). Autotoxic effect of ginsenoside extracts on growth of American ginseng in different medium. China J. Mat. Med..

[55] Cipollini D., Rigsby C.M., Barto E.K. (2012). Microbes as targets and mediators of allelopathy in plants. J. Chem. Ecol..

[56] Pélissier R., Violle C., Morel J.B. (2021). Plant immunity: Good fences make good neighbors?. Curr. Opin. Plant Biol..

[57] Wang P., Kong C.H., Hu F., Xu X.H. (2007). Allantoin involved in species interactions with rice and other organisms in paddy soil. Plant Soil..

[58] Takagi H., Ishiga Y., Watanabe S., Konishi T., Egusa M., Akiyoshi N., Matsuura T., Mori I.C., Hirayama T., Kaminaka H. (2016). Allantoin, a stress-related purine metabolite, can activate jasmonate signaling in a MYC2-regulated and abscisic acid-dependent manner. J. Exp. Bot..

