# Supplementary material for: Cell-Free Extracts of the Ginseng Soil Bacterium Pseudomonas plecoglossicida Promote Suppression of Resistance of American Ginseng (Panax quinquefolius) to Root Rot Caused by Ilyonectria mors-panacis
Source: Biology (Basel). 2024 Aug 29;13(9):671. doi: 10.3390/biology13090671 (PMC11428298; doi:10.3390/biology13090671)
Supplement: Supplementary file 1 [file biology-13-00671-s001.zip › biology-3144592-supplementary.pdf]

Table S1. Bacterial glycoside hydrolase protein sequences obtained from NCBI using the terms "ginsenoside transformation", "metabolism of ginsenoside" and "transform ginsenoside" as queries.

Organism	Isolate	Classification	Protein ID	Function
Bifidobacterium longum	H-1	Actinobacteria	ADY62498	metabolizing Rb1 to CK
Burkholderia sp.	QM04	Proteobacteria	AGA60123.1	ginsenoside transformation activity
Chryseobacterium caeni	KCTC12506	Bacteroidetes	AGA60128.1	ginsenoside transformation activity
Microbacterium esteraromaticum	GS514	Actinobacteria	AEX88467.1	catalyzing conversion of Rb1 to Rd and CK
M. esteraromaticum	KACC16318	Actinobacteria	AEX88466.1	hydrolyzing Rb1 to Rd and 20(*S*)-Rg3
Pseudonocardia sp.	Gsoil1536	Actinobacteria	AGA60129.1	ginsenoside transformation activity
Pseudonocardia sp.	Gsoil1536	Actinobacteria	AGA60134.1	ginsenoside transformation activity
Terrabacter ginsenosidimutans	Gsoil 3082	Actinobacteria	ACZ66247.3	catalyzing conversion of Rb1 to gypenoside XVII, gypenoside LXXV and CK

Table S2. Bacterial glycoside hydrolase protein sequences obtained from NCBI for xylosidase using the term 'ginsenoside hydrolyzing', rhamnosidase using the term '(pseudomonas [Organism]) AND rhamnosidase)' and α-L-arabinofuranosidase using the term '(pseudomonas[Organism]) AND α-L-arabinofuranosidase ' as queries.

Organism	Isolate	Classification	Protein ID	Gene annotation	Function
Bifidobacterium breve	K-110	Actinobacteria	ABX45137.1	β-D-xylosidase	Hydrolyzing Ra1
Pseudomonas aeruginosa	Pa1060	Proteobacteria	RLR70801.1	rhamnosidase	-
Pseudomonas spp.	HMWF010	Proteobacteria	PTT79144.1	α-L-arabinofuranosidase	-

Table S3. Bacterial outer membrane protein sequences obtained from NCBI using the term 'ginsenoside aglycons' as a query.

Organism	Isolate	Classification	Protein ID	Gene annotation	Function
Sphingobacterium multivorum	GIN723	Bacteroidetes	AGP51342.1	Similar to OmpA/MotB	Ginsenoside hydrolysis
Sphingobacterium multivorum	GIN723	Bacteroidetes	AGP51343.1	Similar to TonB-dependent receptor	Ginsenoside hydrolysis

Table S4. Bacterial glycoside oxidoreductase and TAT-pathway signal protein sequences obtained from NCBI using the term 'deglycosylation of ginsenosides' as a query.

Organism	Isolate	Classification	Protein ID	Gene annotation	Function
Rhizobium sp.	GIN611	Proteobacteria	AEX01166.1	Glycoside Oxidoreductase	Ginsenoside deglycosylation
Rhizobium sp.	GIN611	Proteobacteria	AEX01167.1	TAT-pathway signal protein	Ginsenoside deglycosylation
